# Mercurial Treatment in Typhoid Fever

**Published:** 1847-10

**Authors:** 


*Mercurial Treatment in Typhoid Fever.* By M. Serres.—M. Serres begins by stating that, in his opinion, typhoid fever consists in an exanthematous affection of the intestines. The febrile excitement, the diarrhœa, abdominal symptoms, and cerebral manifestations are entirely governed in their progress and intensity by the intestinal eruption. In this respect M. Serres thinks that typhoid fever can be most properly compared with small pox, in which Sydenham has shown that the violence of the malady is always proportioned to the confluence or mildness of the cutaneous eruption. The professor, therefore, concludes that, by keeping the intestinal diseases under control in typhoid fever, the general reaction, its consequence, will be thereby prevented from attaining any dangerous height; and no medicine seems to M. Serres so well calculated to produce this result as mercury. Every second day M. Serres prescribes the following pills:—℞. Hydrarg. sulphureti cum sulphure. gr, xviij.; tragacanthæ, gr. x.; syrup q. s. fiat massa in pilul. iv. dividenda. Every morning inunctions with oz. ij. of mercurial ointment are made upon the abdomen. The treatment is suspended when incipient stomatitis is noticed. Under the influence of mercury, diarrhœa is gradually arrested, the tympanitis reduced or prevented, and, although the average duration of the malady is not diminished, still its violence is much abated.—*London Medical Times.*

